# Compensation for the Variable Cyclic Error in Homodyne Laser Interferometers

**DOI:** 10.3390/s150203090

**Published:** 2015-01-30

**Authors:** Pengcheng Hu, Jinghao Zhu, Xuanbiao Guo, Jiubin Tan

**Affiliations:** Harbin Institute of Technology, D-403 Science Park, 2 Yikuang Street, Harbin 150080, China; E-Mails: hupc@hit.edu.cn (P.H.); 13B901015@hit.edu.cn (J.Z.); 13B901006@hit.edu.cn (X.G.)

**Keywords:** homodyne interferometer, quadrature signal, cyclic error compensation

## Abstract

This paper presents a real-time method to compensate for the variable cyclic error in a homodyne laser interferometer. The parameters describing the quadrature signals of the interferometer are estimated using simple peak value detectors. The cyclic error in the homodyne laser interferometer was then corrected through simple arithmetic calculations of the quadrature signals. A field programmable gate array was utilized for the real-time compensation of the cyclic error in a homodyne laser interferometer. The simulation and experimental results indicated that the proposed method could provide a cyclic error that was fixed without compensation down to a value under 0.6 nm in a homodyne laser interferometer. The proposed method could also reduce the time-varying cyclic error to a value under 0.6 nm in a homodyne laser interferometer, in contrast to the equivalent value of 13.3 nm for a conventional elliptical fitting method.

## 1. Introduction

Homodyne laser interferometers have been widely used for high-precision measurements of displacement because of their simple configuration, high resolution and accuracy, direct traceability to the primary standard of length, *etc.* [1–4]. However, the sub-nanometer performance of a homodyne laser interferometer is often severely limited by a cyclic error, which is usually below 20 nm [5–14]. To meet the requirements for better displacement metrology, interferometer optical and electronic non-linearities, noise and stability well below sub-nanometer values are necessary [15].

Much work has been performed in recent years on the removal of the cyclic error. For example, Heydemann [5], Wu [6], and Eom *et al.* [7–10] elliptically fitted the quadrature signals of a homodyne interferometer and then calibrated the cyclic error using software or analog electrical circuits. All of these approaches have shown superior capability to reduce cyclic error when it is fixed during the measurement.

However, in some applications, the offsets and the amplitudes of the interference signals change during the measurement. In this case, real-time identification of the variable cyclic error model is necessary, and real-time correction is required to address the environmental fluctuations, such as changes in temperature or tilt of a plane mirror reflector or lateral displacement of a corner cube reflector [7,13]. Dai [11] partly recovered the quadrature signals with four peak values to dynamically compensate for the cyclic error. Fan [12] showed that the phase delay error can always be corrected by orthogonal signal compensation. Keem [13] was able to dynamically compensate the AC amplitude error and DC offset error of the distorted quadrature signals, but the phase delay or lack of quadrature still remained as a cyclic error source. These methods are effective but are not sufficiently dynamic to enable high-speed and high-resolution measurements [16].

Kim [17] implemented a digital signal processing method for real-time compensation by estimating the elliptical parameters. The method was implemented by using a field-programmable gate array, which can correct all three types of cyclic error (the offsets error, the amplitudes error, and the quadrature phase delay error). However, this method involved a relatively time-consuming iterative process due to the fact that the determination of fixed points and calculating the value of phase φ are interdependent. In this paper, we propose a real-time approach for the compensation of the cyclic error, with focus on how to improve the estimation of the parameters with enhanced arithmetic operations using an FPGA.

## 2. Cyclic Error in a Homodyne Laser Interferometer

**Figure 1.**
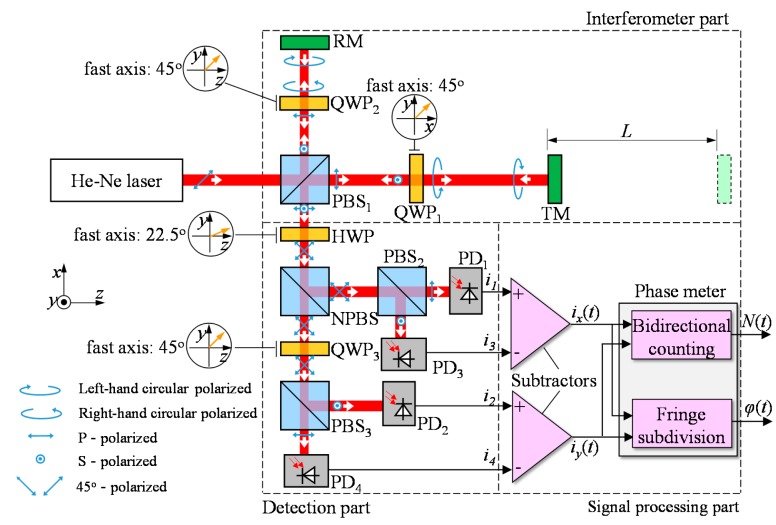
Schematic diagram of the homodyne interferometer with quadrature detection system. Polarizing Beam Splitter (PBS), Quarter-Wave Plate (QWP), Target Mirror (TM), Reference Mirror (RM), Half-Wave Plate (HWP), Non-Polarizing Beam Splitter (NPBS), Photo Detector (PD).

As shown in Figure 1, a homodyne interferometer can be divided into three parts: an interferometer part, a detection part and a signal processing part. In the interferometer part, a 45° linear polarized laser beam was applied to a polarizing beam splitter (PBS) and divided into two beams, one vertically polarized and the other horizontally polarized, which are propagated along the separated arms of the interferometer. In each of the arms, the beam passed through a quarter-wave plate (QWP) twice, and its polarization state was rotated through 90°, so that the beam initially reflected by PBS was then transmitted and propagated to the detection part, and the beam initially transmitted by the PBS was then reflected and propagates to the detection part. In the detection part with a half wave-plate (HWP), a QWP, a non-polarizing beam splitter (NPBS) and two PBSs, interference signals, *i*_1_, *i*_2_, *i*_3_ and *i*_4_, were detected by the four photo detectors, and they had the phases of 0°, 90°, 180° and 270°, respectively. In the signal processing part, the two quadrature signals were obtained by subtracting *i*_1_ from *i*_3_ and *i*_2_ from *i*_4_, and then they were converted into a displacement through bidirectional counting and fringe subdivision.

**Figure 2.**
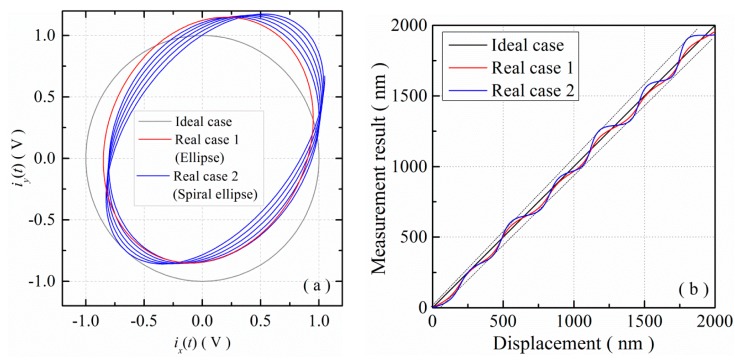
Trajectories of quadrature signals and displacement measurements in ideal and real cases: (**a**) trajectories and (**b**) measurements.

It could be observed from Equation (1) below that under the ideal conditions, two quadrature signals, *i_x_*(*t*) and *i_y_*(*t*), should have the same AC amplitude but no DC offset and an exact phase difference of 90°. As a result, the Lissajous trajectory of two ideal quadrature signals had a zero-centered circular shape, as shown in Figure 2, and phase φ(*t*) could be acquired by using Equation (2):

(1) $$\begin{array}{cc} i_{x}(t)=A\cos\varphi (t), & i_{y}(t)=A\sin\varphi (t) \end{array}$$

(2) $$\varphi (t)=\{\begin{array}{cc} \arctan[i_{x}(t)/i_{y}(t)]+2\pi \times N(t), & i_{x}(t)>0\\ \arctan[i_{x}(t)/i_{y}(t)]+\pi +2\pi \times N(t), & i_{x}(t)\leq 0 \end{array}$$

where *A* is the AC amplitude and *N*(*t*) is the output value of the bidirectional counter. When the target mirror moved by distance *L* during the time period of [*t*_0_, *t*_1_], the phase shift in interference signals, Δφ, was proportional to the displacement of the target mirror, which could be acquired using the following equation:

(3) $$L=\frac{\Delta \phi }{4\pi }\times \lambda =\frac{\phi (t_{1})-\phi (t_{0})}{4\pi }\lambda$$

where *λ* was the laser wavelength.

However, as observed from Equation (4) below, in reality, the quadrature signals have not only an amplitude difference and a DC offset but also a phase delay [10] because of the misalignment of optical elements, the imperfections in optical elements and electronic circuits, or the improper movement of the target mirror [5–10]. As a result, the Lissajous trajectory of the two quadrature signals was distorted from an ideal circle, and cyclic error would occur when the ideal model in Equation (2) was utilized to calculate the phase of real quadrature signals:

(4) $$\begin{array}{cc} i_{x}(t)=A_{x}+B_{x}\cos\phi (t), & i_{y}(t)=A_{y}+B_{y}\sin[\phi (t)+\delta ] \end{array}$$

where *A_x_* and *A_y_* are the DC offsets, *B_x_* and *B_y_* are the different AC amplitudes, and δ is the phase delay from 90°. The cyclic error of the phase measurements could be expressed as:

(5) $$NL=\arctan\frac{A_{y}+B_{y}\sin[\varphi (t_{1})+\delta ]}{A_{x}+B_{x}\cos\varphi (t_{1})}-\arctan\frac{A_{y}+B_{y}\sin[\varphi (t_{0})+\delta ]}{A_{x}+B_{x}\cos\varphi (t_{0})}-[\varphi (t_{1})-[\varphi (t_{0})]$$

It could be observed from Equation (1) below that under the ideal conditions, two quadrature signals, *i_x_*(*t*) and *i_y_*(*t*), should have the same AC amplitude but no DC offset and an exact phase difference of 90°. As a result, the Lissajous trajectory of two ideal quadrature signals had a zero-centered circular shape, as shown in Figure 2, and phase φ(*t*) could be acquired by using Equation (2).

The real quadrature signals in Equation (5)*A_x_*, *A_y_*, *B_x_*, *B_y_* and δ, are different for different homodyne interferometers, and even in the same homodyne interferometer, these parameters tend to be time-varying [9,18]. In this case, the Lissajous trajectory of real quadrature signals has an elliptical shape or a spiral elliptical shape, as shown in Figure 2, and the cyclic error will be different for individual homodyne interferometers and will vary as time elapses, even in the same homodyne interferometer. The cyclic error model need to be real-time identified and compensated.

## 3. Real-Time Compensation Method

**Figure 3.**
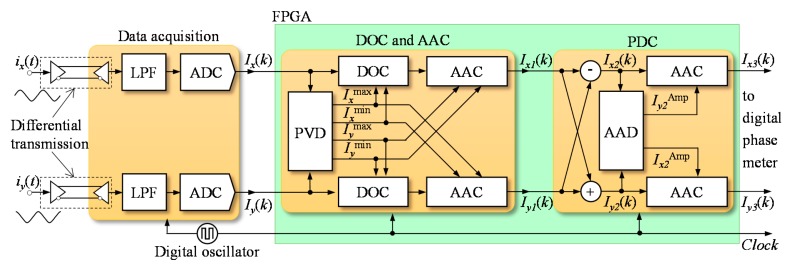
Block diagram of the approach for real-time compensation of cyclic error. A schematic of the proposed real-time cyclic error compensation method: LPS (Low-Pass Filter), ADC (Analog-to-Digital Convertor), DOC (DC Offset Correction), AAC (AC Amplitude Correction), PVD (Peak Value Detection), PDC (Phase Delay Correction) and AAD (AC Amplitude Detection).

As shown in Figure 3, the proposed method is composed of three systems: a data acquisition system, a DC offset and AC amplitude difference correction system and a phase delay correction system. These three systems were synchronized by the same high-speed clock and worked in parallel with each other.

The quadrature signals were transmitted into the data acquisition system through the differential transmission wires with high anti-disturbance. In the data acquisition system, the quadrature signals were preprocessed with a low-pass filter and converted into digital data through two high-speed analog-to-digital convertors (ADCs). The processed signals can be expressed as:

(6) $$\begin{array}{ccc} I_{x}(k)=i_{x}(kT_{s}), & I_{y}(k)=i_{y}(kT_{s}), & k=0,1,2\ldots \end{array}$$

where *T*_s_ is the sampling period of ADCs.

In the DC offset and AC amplitude difference correction system, the fast peak detection module could be used to dynamically check the peak values of *I_x_*(*k*) and *I_y_*(*k*). Upon completion of this correction, there was still a phase delay between the corrected quadrature signals, *I_x1_*(*k*) and *I_y1_*(*k*), respectively.

**Figure 4.**
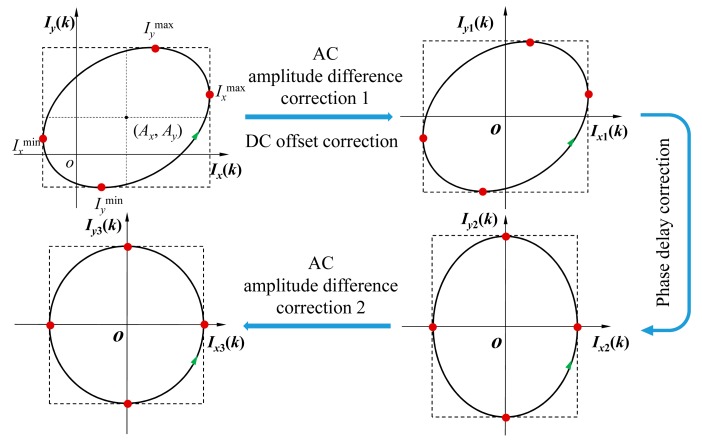
Schematic diagram of the compensation process.

The vector summation and subtraction modules in the phase delay correction system were used to correct the phase delay. The AC amplitude difference detection and correction modules were utilized to compensate for the new AC amplitude difference between two quadrature signals, which resulted from the vector summation and subtraction operations. Finally, new digital quadrature signals *I_x_*_3_(*k*) and *I_y_*_3_(*k*), without AC amplitude difference, DC offset, or phase delay, were transmitted into the digital phase meter for the measurement of displacement without cyclic error. The basic principle of the compensation method proposed is shown in Figure 4.

### 3.1. Correction of the DC Offset and AC Amplitude Difference

**Figure 5.**
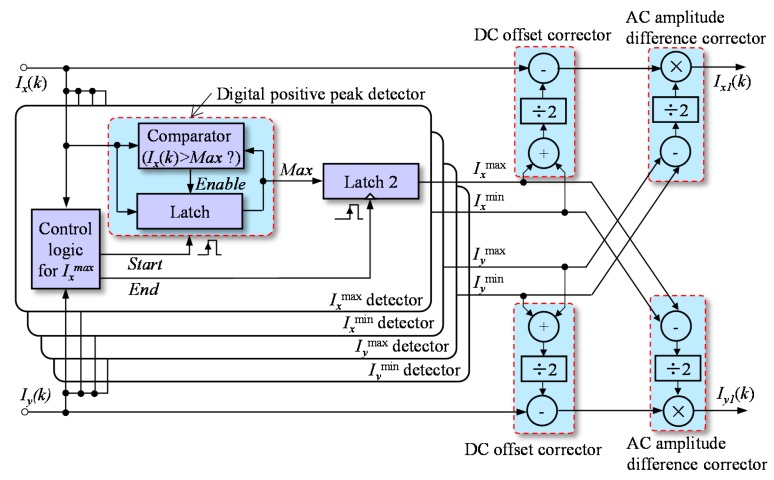
Correction of the DC offset correction and AC amplitude difference.

As shown in Figure 5, four peak detectors were utilized to capture the four peaks of the two quadrature signals, *I_x_*^max^, *I_x_*^min^, *I_y_*^max^ and *I_y_*^min^, which could be expressed as:

(7) $$\begin{array}{cc} I_{x}^{\max}=A_{x}+B_{x}, & I_{x}^{\min}=A_{x}-B_{x} \end{array}$$

(8) $$\begin{array}{cc} I_{y}^{\max}=A_{y}+B_{y}, & I_{y}^{\min}=A_{y}-B_{y} \end{array}$$

The DC offset and AC amplitude could be dynamically corrected by two pairs of correctors through simple arithmetic operations, which could be expressed as:

(9) $$I_{x1}(k)=\frac{I_{y}^{\max}-I_{y}^{\min}}{2}\times [I_{x}(k)-\frac{I_{x}^{\max}-I_{x}^{\min}}{2}]=B_{y}B_{x}\cos[\phi (kT_{s})]$$

(10) $$I_{y1}(k)=\frac{I_{x}^{\max}-I_{x}^{\min}}{2}\times [I_{y}(k)-\frac{I_{y}^{\max}-I_{y}^{\min}}{2}]=B_{x}B_{y}\sin[\phi (kT_{s})+\delta ]$$

This correction is performed with each pair of digitalized quadrature signals, and the parameter values will be updated whenever one of the peak values is newly updated. Upon completion of this correction, only the phase delay remains in the two corrected quadrature signals, *I_x_*_1_(*k*) and *I_y_*_1_(*k*).

As shown in Figure 5, besides a common digital positive peak detector, detector *I_x_*^max^ also contained a digital latch and control logic. If the current input signal of the digital positive peak detector, *I_x_*(*k*), was greater than its output signal *Max*, *Max* would then be updated and kept as *I_x_*(*k*). The control logic had two output signals, *Start* and *End*. At the rising edge of *Start*, output signal, *Max*, of the digital positive peak detector was reset to zero. At the rising edge of *End*, *Max* was captured by the latch, and the captured value became the output of detector *I_x_*^max^.

**Figure 6.**
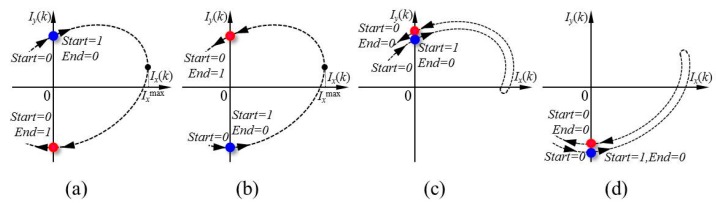
Detection of peak value for *I_x_^max^*. (**a,b**) *I_x_^max^* was detected successfully; (**c,d**) *I_x_^max^* was not detected.

As shown in Figure 6a, *Start* changed from 0 to 1 to reset the digital positive peak detector and *End* changed from 1 to 0 when the trajectory of two quadrature signals traveled from the 2nd quadrant section to the 1st quadrant section. *I_x_*^max^ would be successfully captured by the detector when the trajectory traveled across the 1st and 2nd quadrant sections. At the end of traversing from the 4th to the 3rd quadrant section, *End* changed from 0 to 1 to update the detector to output *I_x_*^max^ as a result. The same procedure was valid for the trajectory shown in Figure 6b.

As shown in Figure 6c, at the end of the traversal from the 2nd to the 1st quadrant section, the trajectory did not continue its travel to the 4th and 3rd quadrant sections, as mentioned above, but returned from the 1st to the 2nd quadrant section once again, which made it difficult to determine whether the real *I_x_*^max^ was obtained. To avoid such a vague *I_x_*^max^, *End* would remain to be 0 if the trajectory missed the progress of going across the 1st quadrant section and the 4th quadrant section to the 3rd quadrant section, so that the detector would not update an improper *I_x_*^max^. The same procedure was valid for the trajectory shown in Figure 6d. The control logic for detectors *I_x_^min^*, *I_y_^max^*, and *I_y_^min^* is similar to that mentioned above regarding detector *I_x_*^max^.

The correction method proposed was unique regarding the following two aspects: (1) there was no iterative process in our method, which enables the model to save more time; (2) instead of floating-point division, the correction of the AC amplitude difference was accomplished only through fixed point multiplication operations, which made the calculation efficient and easy to perform using an FPGA chip.

### 3.2. Correction of the Phase Delay

**Figure 7.**
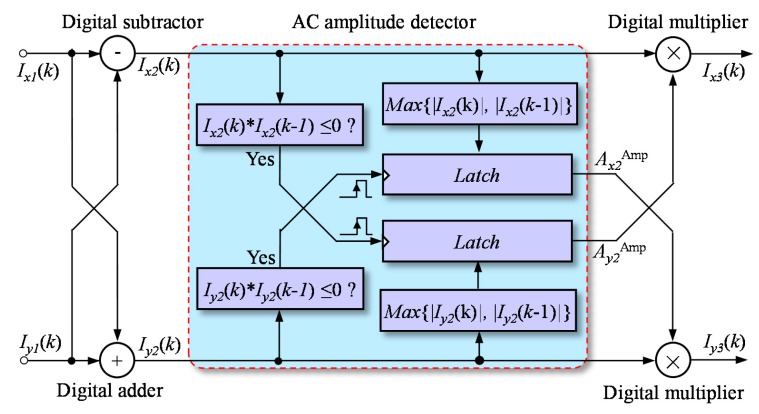
Correction of phase delay. The phase delay error was corrected through vector subtraction and summation operations, and the amplitude correction module was utilized to balance the difference in amplitude resulting from the vector subtraction and summation.

As shown in Figure 7, the phase correction model was mainly composed of a digital subtractor and a digital adder, an AC amplitude detector and two digital multipliers. The vector summation and subtraction operations were performed *via* a digital adder and a digital subtractor. The outputs of the adder and subtractor could be expressed as:

(11) $$\begin{array}{c} I_{x2}(k)=I_{x1}(k)-I_{y1}(k)=2B_{x}B_{y}\sin(\pi /4-\delta /2)\cos[\phi (kT_{s})+\pi /4+\delta /2]\\ I_{y2}(k)=I_{x1}(k)-I_{y1}(k)=2B_{x}B_{y}\cos(\pi /4-\delta /2)\sin[\phi (kT_{s})+\pi /4+\delta /2] \end{array}$$

The new quadrature signals, *I_x_*_3_(*k*) and *I_y_*_3_(*k*), had an exact phase difference of 90°. As a result, the vector subtraction and summation operations could eliminate the phase delay of quadrature signals. However, from Equation (11), the vector operations caused the same phase offset for the new quadrature signals and caused their amplitudes to be different. The displacement measurement in a homodyne interferometer was calculated with the change in phase; as a result, the phase offset in Equation (11) would not have any effect on the measurement of displacement.

An AC amplitude detector and two digital multipliers were used to balance the amplitudes of *I_x_*_2_(*k*) and *I_y_*_2_(*k*). Because there was no DC offset or phase delay in *I_x_*_2_(*k*) and *I_y_*_2_(*k*), their AC amplitudes could be detected in a simple way. According to Equation (11), the AC amplitude of *I_x_*_2_(*k*) could be achieved when *I_y_*_2_(*k*) was approximately zero, and the AC amplitude of *I_y_*_2_(*k*) could be achieved when *I_x_*_2_(*k*) was approximately zero. The function of the AC amplitude detector could be expressed as:

(12) $$\begin{array}{c} I_{x2}^{\text{Amp}}=\max\{|I_{x2}(k)|,|I_{x2}(k-1)|\},ifI_{y2}(k)\times I_{y2}(k-1)\leq 0\\ I_{y2}^{\text{Amp}}=\max\{|I_{y2}(k)|,|I_{y2}(k-1)|\},ifI_{x2}(k)\times I_{x2}(k-1)\leq 0 \end{array}$$

From Equation (11), the AC amplitudes of *I_x_*_2_(*k*) and *I_y_*_2_(*k*) could be expressed as:

(13) $$\begin{array}{c} I_{x2}^{\text{Amp}}=2B_{x}B_{y}\sin(\pi /4-\delta /2)\\ I_{y2}^{\text{Amp}}=2B_{x}B_{y}\cos(\pi /4-\delta /2) \end{array}$$

These AC amplitudes respectively multiplied *I_y_*_2_(*k*) and *I_x_*_2_(*k*) as:

(14) $$\begin{array}{c} I_{x3}(k)=I_{y2}^{\text{Amp}}\times I_{x2}(k)=B\cos[\phi (kT_{s})+\pi /4+\delta /2]\\ I_{y3}(k)=I_{x2}^{\text{Amp}}\times I_{y2}(k)=B\cos[\phi (kT_{s})+\pi /4+\delta /2] \end{array}$$

where *B* = 2(*B_x_B_y_*)^2^sin(*π*/2 − δ). The new signals, *I_x_*_3_(*k*) and *I_y_*_3_(*k*), had the same AC amplitude, zero DC offset and an exact phase difference of 90°. As a result, the phase calculation results obtained using Equation (2) were free from cyclic error, and as a result, the displacement measurements of a homodyne interferometer were free from cyclic error.

The compensation of cyclic error was performed with simple hardware in FPGA through simple arithmetic calculations. The parameters used in the calculation models were dynamically obtained using several digital peak and amplitude detectors. It was therefore possible to realize the real-time identifying and compensation of the cyclic error in homodyne laser interferometers.

### 3.3. Numerical Simulation and Analysis

The effectiveness of the method proposed was verified through numerical simulations. The parameters used during the simulations are as follows: *A_x_* = −0.15 V, *B_x_* = 0.7 V, *A_y_* = −0.1 V, *B_y_* = 0.8 V, φ(*t*) = 400π*t*, *T*_s_ = 0.1 ms, and δ = 10°. To simplify the calculations, the measurement errors of ADCs, the digital phase meter, and the finite word length effect were not taken into account during the numerical simulations.

**Figure 8.**
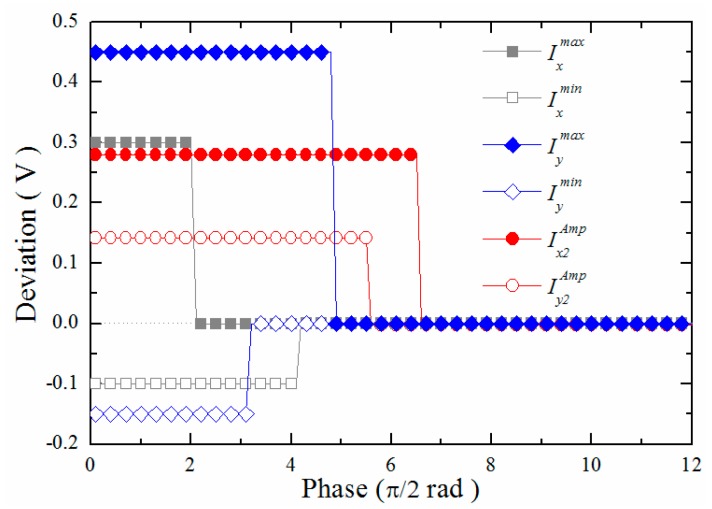
Differences between the parameters obtained using detectors and the ideal parameters.

To evaluate the peak value detectors and the amplitude detectors, the differences between the estimated values using several detectors and the ideal parameter values were not updated until the quadrature signals covering a π phase angle were obtained. As shown in Figure 8, the differences decreased within the phase angle 3π, and all of the final deviations were less than 2.5 mV. These parameters were updated when the raw quadrature signals, *I_x_*(*k*) and *I_y_*(*k*), or the signals after the vector calculation, *I_x_*_3_(*k*) and *I_y_*_3_(*k*), are passed through the coordinate axis. The quadrature signals were then recovered using the updated parameters.

**Figure 9.**
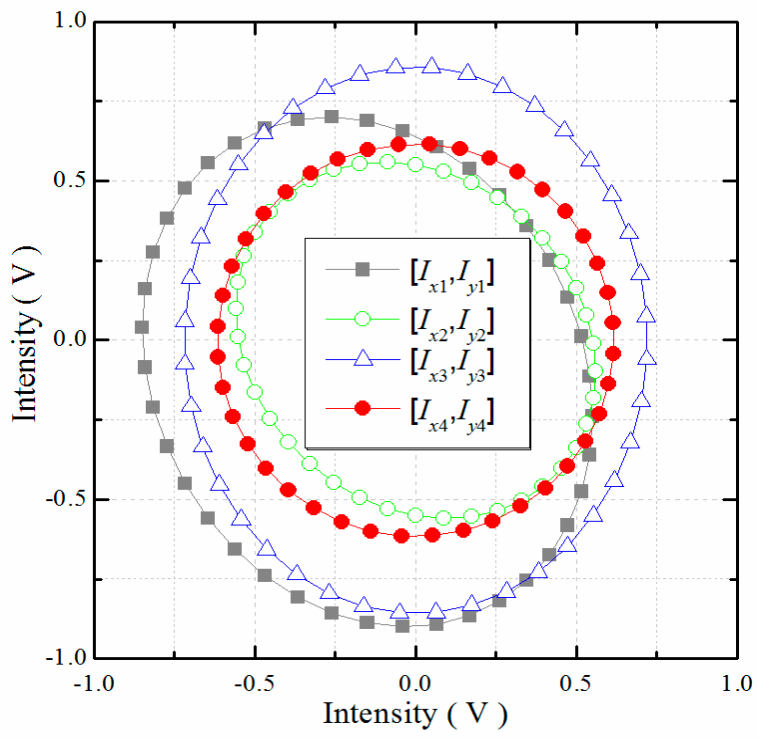
Lissajous trajectories of several pairs of quadrature signals during cyclic error compensation using the method proposed.

As shown in Figure 9, DC offset error, AC amplitude error, and phase delay error were found in *I_x_*(*k*) and *I_y_*(*k*). After the primary compensation, only a phase delay error was found in *I_x_*_1_(*k*) and *I_y_*_1_(*k*). As a result of the vector calculation, AC amplitude error occurred in *I_x_*_2_(*k*) and *I_y_*_2_(*k*) once again. Finally, the DC offset error was found to almost vanish, and the AC amplitude error or phase delay error almost disappeared in *I_x_*_3_(*k*) and *I_y_*_3_(*k*).

**Figure 10.**
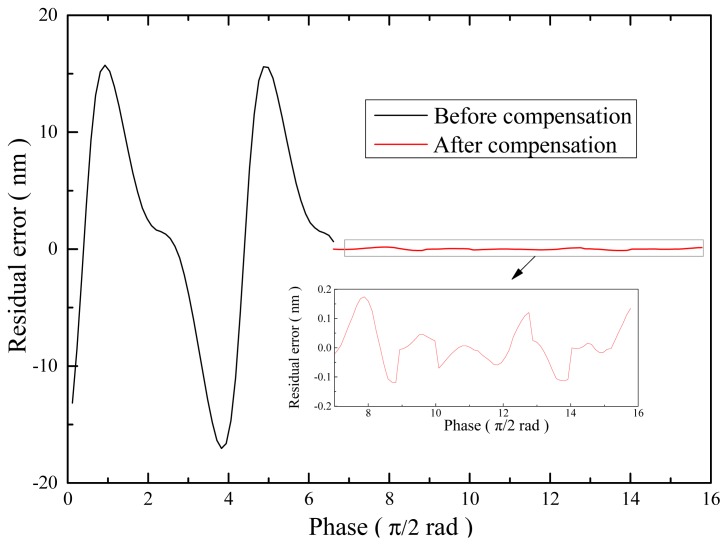
Cyclic errors before and with compensation using the proposed method.

As shown in Figure 10, without compensation, the cyclic error was approximately 32 nm. After the compensation using the method proposed, the residual error decreased to approximately 0.35 nm. Because Gaussian noise has been introduced to the signals during simulation, the compensated error is discontinuous. For our algorithm, noise fluctuations can have effect on the capturing of the peaks of the quadrature signals for identifying of the correction coefficients in each cycle, which can lead to the over-compensation or under-compensation of the cyclic error. With a deviation of approximately 0.1 nm, the discontinuous residual error can be reduced by a Kalman filter in feedback control systems [19].

## 4. Experimental Results

To verify the effectiveness of the method proposed, experiments were also conducted using a homemade quadrature signal processing board (HQSPB). The HQSPB was implemented *via* two 14-bit ADCs operating at the maximum sampling rate of 65 MHz, with the developed cyclic error compensating module and a digital phase meter sharing the same FPGA of the compensation module. All three parts are synchronized by a 50-MHz digital oscillator. The phase meter operation is performed *via* a fast look-up table (LUT) acting 4096 times to interpolate the 2π phase angle [20]; its resolution amounts to 0.077 nm in the case of a single-pass plane mirror interferometer and fringe subdivision noise for acting 4096 times is approximately 0.038 nm. The loop time for a complete procedure after cyclic error compensation is 500 ns, and the cyclic error compensation process except for the ADC process took 160 ns. The update rate of HQSPB can be programmed up to 10 MHz.

### 4.1. Compensation of Cyclic Error at Low Velocity

**Figure 11.**
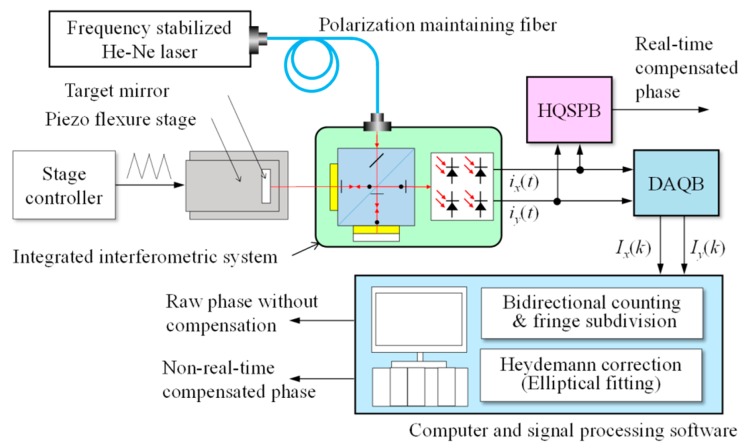
Schematic diagram of the experimental setup: HQSPB (Homemade Quadrature Signal Processing Board) and DAQB (Data Acquisition Board).

**Figure 12.**
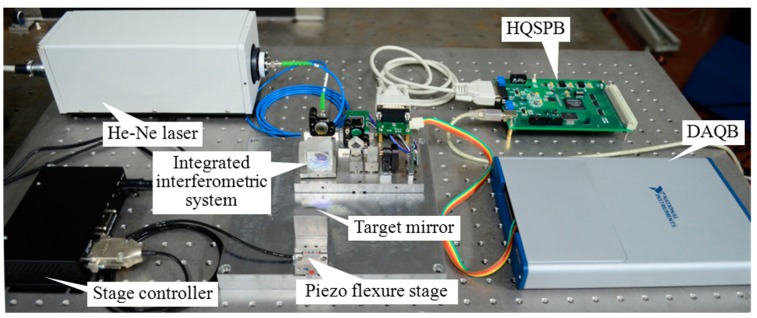
Experimental setup for the compensation of cyclic error.

As shown in Figures 11 and 12, a He-Ne laser whose frequency was stabilized at 633 nm was utilized as the laser source and was coupled to a commercial integrated interferometer system (S2800, Harbin HUE Ltd., Harbin, China) through a polarization maintaining fiber. The target mirror of the interferometer was attached to a one-axis piezo flexure stage (P-753.2CD, Physik Instrument, Karlsruhe, Germany), which was controlled by a high-speed stage position controller (PI E-709.CP, Physik Instrument). A pair of quadrature signals from the integrated interferometer system was transmitted into the HQSPB and a 16-bit resolution data acquisition board (USB 6356, National Instruments, Austin, TX, USA) at the same time. The HQSPB could be used to obtain the real-time compensated phase information from the quadrature signals, while the data acquisition board and a computer were used to obtain both the raw phase information without cyclic error compensation and the non-real-time compensated phase information using the conventional elliptical fitting method [5].

First, the stage was driven by an open loop in a 0.1-Hz triangular wave with 4-V amplitude, which will result in a displacement of approximately 1 μm. During this process, the computer gathered the raw phase without compensation, the non-real-time compensated phase using the conventional elliptical fitting method, and the real-time compensated phase using the proposed method. In addition, the stage will not move linearly due to the nonlinear characteristic of a piezo actuator. To remove this nonlinearity, the cyclic error was calculated by fitting the calculated displacement with a third-order polynomial [10,13].

Note that currently, most of the laser interferometers are unable to accurately measure when the displacement is less than one phase cycle. In this case, self-calibration is required before measuring. To accomplish the self-calibration, the target mirror should be moved back and forth more than half of the wavelength to achieve the maximum values of the fringes. In fact, in this article, the first cycle is a calibration process.

Moreover, noise sensitivity is important for this work. In the experiment, the signal-to-noise ratio of the interference signals is about 61 dB, and the signal-to-noise of the ADC is 66 dB with an effective number of bits 10.74, so the maximum permissible noise level is 0.9‰ (SNR = 61 dB) for the interferometry measurement. Then the deviation of the peak capturing for the compensation of cyclic error is 0.9‰, which will result in a residual error of approximately 0.37 nm in length with a numerical simulation refer to Section 3.3.

**Figure 13.**
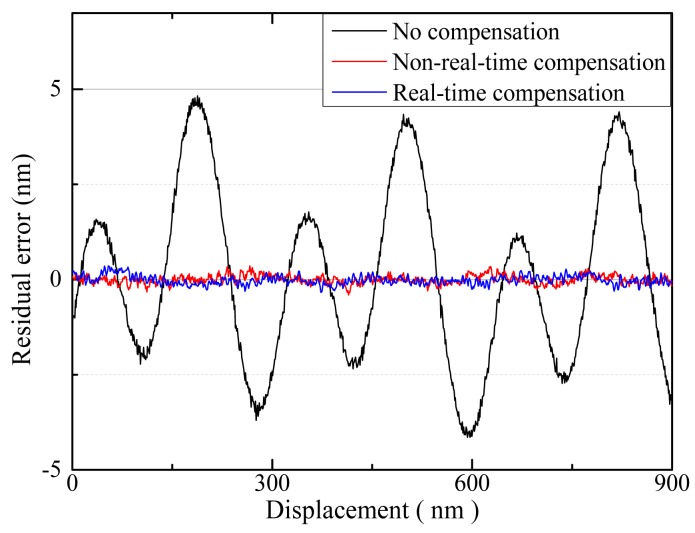
Comparison of the experimentally acquired cyclic error.

As shown in Figure 13, the cyclic error of the homodyne interferometer was approximately 8.35 nm without compensation. Both non-real-time compensation and real-time compensation methods could be used to keep the cyclic error of the homodyne interferometer under 0.52 nm, *i.e.*, approximately 1/16 of the original value. This residual error is greater than we estimated, which because that the residual error contains the high-order terms of the cyclic error in a homodyne interferometer [17], the electrical noise, the improper angular motion of the stage and the instability of the refractive index of air [13,18].

However, the compensation method fails to exhibit an advantage compared to the non-real-time method in terms of the dynamic properties. This lack of advantage was observed because in this experiment, the moving velocity of the target mirror was set as 0.2 μm/s and there was abundant time for the non-real-time method to complete complex computations such as elliptical fitting *via* a least-squares method.

### 4.2. Compensation of Cyclic Error at High Velocity

To verify the dynamic performance of the proposed method, another experimental setup and new test methods were developed and used.

During this experiment, the simulated interference signals produced by an arbitrary wave generator (AWG 5012C, Tektronix, Beaverton, OR, USA) were used to provide better accuracy and programmability and to eliminate other sources of systematic errors [16]. The frequencies of two simulated interference signals were kept invariant for each test. The simulated signals could therefore be seen as the quadrature signals from a real homodyne interferometer system, where the target mirror was moving accurately at a constant velocity. Furthermore, the simulated signals were transmitted to the HQSPB, and the measurement results of HQSPB with a fixed time interval of 100 ns and the measurement results were fitted for the time elapsed to a line. As a result, the residuals in the linear fitting process were the cyclic error after the real-time compensation.

**Figure 14.**
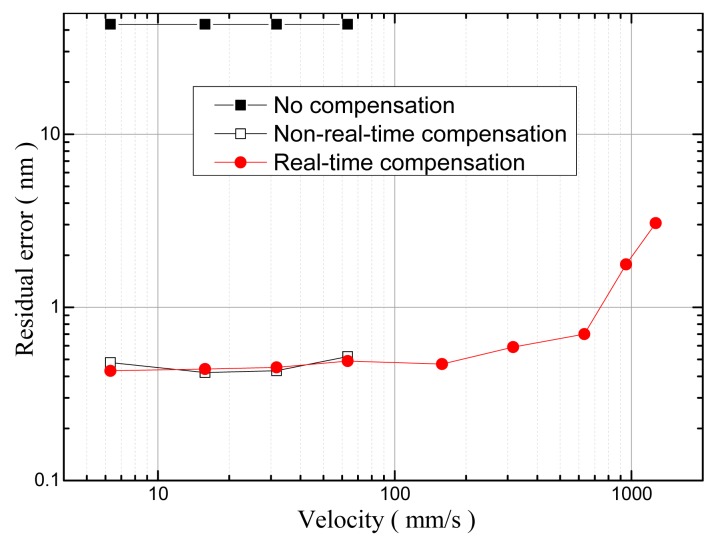
Cyclic error *vs.* velocity of the target mirror, the residual error was measured after a linear fit. Note that when the simulation velocity of the target mirror was greater than 100 mm/s, no experimental results were obtained regarding the cyclic error without compensation or the cyclic error with non-real time compensation because the maximum sampling rate of DAQB was limited to 1.2 MHz.

As shown in Figure 14, the residuals existed after real-time cyclic error compensation when the simulated signals were set as:

(15) $$i_{x}(t)=0.1V+0.5V\times \cos(2\pi ft),i_{y}(t)=-0.1V+0.8V\times \sin(2\pi ft+\pi /18)$$

where *f* = 2*v*/λ is the Doppler frequency. By setting different *f* values, the velocity of the target mirror movement, *v*, could be simulated. As shown in Figure 14, the cyclic error without compensation was 43.08 nm, the non-real-time compensation could suppress the cyclic error to less than 0.6 nm when the target mirror was moving at a velocity in the range between 6.3 mm/s and 63.3 mm/s, and the method proposed could suppress the cyclic error to less than 0.6 nm even when the target mirror was moving at a velocity in the range between 6.3 mm/s and 633 mm/s. However, after the real-time compensation, the residual error increased to 1.77 nm when the velocity was 949.5 mm/s, and it increased to 3.07 nm rapidly when the velocity was 1266 mm/s. This result was obtained because when the frequency of quadrature signals was greater than 3 MHz, the peak value detection modules and amplitude detection modules in the HQSPB were both synchronized by a 50 MHz clock, and they could not accurately capture the peak values of the quadrature signals.

**Figure 15.**
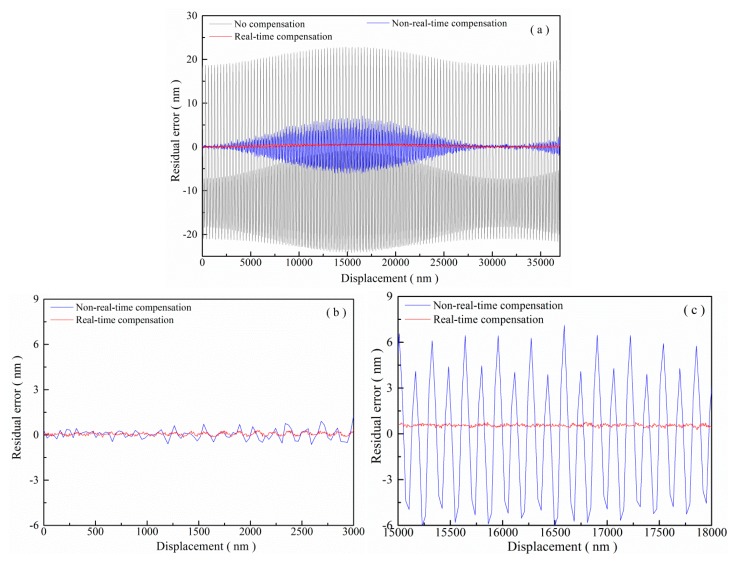
Experimental results of cyclic error compensation with simulated interference signals: (**a**) complete experimental results; (**b**) and (**c**) details of experimental results.

To verify the real-time cyclic error compensation method when the cyclic error is time-varying, all parameters except for the frequency of quadrature signals were programmed to be time-varying, and then the cyclic error after real-time compensation was calculated. As shown in Figure 15, residuals after the real-time cyclic error compensation were found when the simulated signals are set as:

(16) $$\begin{array}{c} i_{x}(t)=A_{x}(t)+B_{x}(t)\cos(2\pi \times 100\text{kHz}\times t)\\ i_{y}(t)=A_{y}(t)+B_{y}(t)\sin[2\pi \times 100\text{kHz}\times t+\delta (t)] \end{array}$$

where the time-varying parameters periodically change by 5% at a frequency of 1 kHz, and the parameters could be expressed as:

(17) $$\begin{array}{c} A_{x}(t)=0.1V+0.005V\times \sin(2\pi \times 1\text{kHz}\times t)\\ B_{x}(t)=0.5V+0.025V\times \sin(2\pi \times 1\text{kHz}\times t)\\ A_{y}(t)=-0.1V+0.005V\times \sin(2\pi \times 1\text{kHz}\times t)\\ B_{y}(t)=0.8V-0.04V\times \sin(2\pi \times 1\text{kHz}\times t)\\ \delta (t)=\pi /18+\pi /360\times \sin(2\pi \times 1\text{kHz}\times t) \end{array}$$

As shown in Figure 15, when the parameters of the simulated signals changed, the cyclic error without compensation varied from 0.6 nm to 13.3 nm, and the residual error after real-time compensation was kept at 0.6 nm. The result occurs because the real-time method can update its estimates of the cyclic error parameters in real time, while the non-real-time model was offline and fixed. The residual error after real-time compensation might be caused by the electrical noise in the arbitrary wave generator and the HQSPB, the errors from digital peak value detection, the amplitude detection, *etc.* Finally, a slight drift of 0.5 nm in the measurement results of Figure 15c was also found after real-time compensation. This drift can be explained by Equation (14), *i.e.*, any drifts in δ(t) will cause corresponding drifts in the measurement results of the phase and the displacement.

## 5. Conclusions

In this publication, a real-time method was presented for compensation of the cyclic error in a homodyne laser interferometer through simple arithmetic calculations of the quadrature signals. The simulation and experimental results indicated that the compensation method proposed is robust for the variable cyclic error model. The method could be used to estimate the time-varying parameters of the real quadrature signals to perform correction in real time and to precisely compensate for the cyclic error in homodyne laser interferometers. As shown in the experimental results above, the amplitude difference, DC offset and phase delay in homodyne laser interferometers could be corrected in a loop time of 160 ns. In homodyne laser interferometers, under both low and high velocity conditions, the cyclic error could be reduced to a value below 0.6 nm. The proposed method could also be used to compensate for the cyclic error in gratings, grating interferometers, *etc.*
